# Clinical Reports

**Published:** 1860-12

**Authors:** N. S. Davis

**Affiliations:** Professor of Practical Medicine and of Clinical Medicine


### CLINICAL REPORTS.

### MERCY HOSPITAL.

Service of Prof. N. S. DAVIS, M. D., Prof. of Practical Medicine and of Clinical Medicine.

(Collated by FRANK W. REILLY, Senior Class Med. Depart. Lind University.)

### *Hypertrophy with Dilatation of the Left Auricle—Result: Thickening of Mitral Valve, Œdema and Pulmonary Congestion.*

The patient, a German Catholic clergyman, *ætat* about 35, was admitted into the Hospital on the 19th November, from one of the interior towns of the State. Appearances before the class—anæmic; complexion sallow, bloodless; countenance anxious; pulse full, strong, irregular and intermittent; inferior extremities œdematous. About two years since had a severe attack of fever (probably typhoid, from description), to which he refers his present condition.

Prefacing with a brief review of the pathology of hypertrophy, its causes, conditions and effects, the lecturer proceeded to remind the class of the physiology and anatomy of the heart, to which, in this case, attention was specially attracted by the general symptoms and appearances:

As you are aware, gentlemen, the lining membrane of the heart—the *endocardium*—enters into the composition of the cardiac valves and orifices, and, like the pericardium, consists of two layers, an external, fibrous, and an internal, serous, one. Now the result of an infiltration, supervening upon an inflammatory attack of whatever nature, might be a deposit in the texture of the valves, which deposit becoming organized, would occasion a thickening; the patient recovers from his inflammatory attack, his acute rheumatism, or fever, and to all appearances is perfectly well. But, gentlemen, should the deposit fail to be absorbed, and the valve or orifice continue thus impeded by a surplus of matter, fibrous or osseous, what is the consequence? Why, in two, four or six months your patient begins to notice a difficulty in his breathing; he is less able to undergo active physical exertion, running up a flight of stairs "puts him out of breath," as the saying is; the urinary secretion diminishes, there is an excess of phosphatic and lithic acid salts, the quality of the blood is impaired, derangement of the digestion, loss of strength, œdema and general enfeeblement are the results. Now it is necessary to know—not less essential to your own success as practitioners, than to the welfare of your patients—that cardiac disease, either temporary or permanent, is the not uncommon termination of a large proportion of, more especially acute rheumatic attacks. And it is necessary to remember this, not because your patient is in immediate danger,—for, as I have already hinted, the cardiac irritation in many cases is only temporary—but in others it becomes chronic and finally fatal. With this knowledge you will see the propriety of carefully watching, during the progress of an acute attack of inflammatory fever, the condition of the heart's action. I would advise you to auscultate frequently—auscultate at least every second day during the active stage of the disease—and should you detect any evidence of cardiac disease supervening, direct your treatment to it at once, and do not discontinue the use of alteratives and other remedies tending to promote absorption, until all abnormal signs are removed. All cases of valvular disease of the heart, however, are not attributable to rheumatism, though by far the greater portion are. In typhoid or continued fever, we not unfrequently find red, congested patches upon the heart, showing that infiltration might have taken place, as, I doubt not, it did in the case before us. In these cases, however, we should suspect the progress of the disease would be less rapid than in cases resulting from the former more common cause. And we so find it, manifesting itself slowly and insidiously. Thus in this patient, two years had elapsed before the development of his present symptoms.

In investigating all cases of heart disease, you will be careful to thoroughly distinguish between mere functional disturbance and true organic disease, the result of actual organic change. In some of the most distressing cases of cardiac trouble I have ever seen, there was no organic change—no change of structure whatever. I remember one patient particularly, who could not sit down and listen to a detail of these symptoms, without such a tumultuous throbbing of his heart, dyspnœa and fainting, as would lead to the belief that he was actually about to die. Such cases are met with quite frequently, in both sexes, and yet without any structural change at all. In distinguishing them, you will find that in mere functional disorder, the mental emotions have much to do with the symptoms, and thus the paroxysms will be irregular—as apt, or more so, to occur to the patient while lying in bed, quiet, at night, as when about his ordinary business in the day-time. And there will be times when he will be as free from any symptom of heart disease as you or I. But agitate him by any unpleasant news, or sudden mental excitement, or over-load his stomach with a too hearty meal, and you have at once all the symptoms of functional disease of the heart.

In organic disease, on the contrary, the condition of the mind has less controlling effect; while a given physical exertion will produce a recurrence or increase of the paroxysm, be that exercise pleasant and agreeable or otherwise. We possess a more certain means of diagnosis, however, by auscultation, and by careful study of the indications thus afforded, we may with perfect certainty distinguish between functional disturbance and structural disease. You are already aware that the heart's action has three characteristics—the impulse; a first—*long*—and second—*short*—sound; and that when these are performed normally with relation to each other, the rhythm, as it is termed, is said to be normal. The apex of the heart, forced against the ribs, synchronously with the first sound—the systole, as it is called, after which we have a pause, then the short sound, or diastole, then an interval longer than the first, followed by the impulse, systole, pause, &c., and this we have seventy-five times a minute in the healthy condition. Now, no functional or nervous disorder will interfere with these sounds to modify their character or the relative duration of sound and interval—that is, during the interval between the distressing symptoms before referred to, we will find the rhythm of the heart normal and undisturbed. In anæmia we may have the first sound, or systole, accompanied by a soft blowing bellows sound—the *bruit de soufflet* of the French—and this may, in some degree, complicate our diagnosis; but if we remember that the anæmic bellows sound is soft, and not rough, harsh, rasping or filing, and may further be distinguished in the neck, over the sub-clavian artery, we can proceed with our diagnosis. In organic disease we will find the rhythm perverted and unnatural; the first and second sound modified in various ways; the systole lengthened or shortened and changed in character, or the diastole, or both; or both merged into one sound: we may have rasping, filing or sawing sounds, a variety as extensive as the fancy of the listener chooses to recognize. And these sounds will depend upon alterations in the structure of the valves or orifices, and from their location, character and other indications, we are enabled to locate with a very considerable degree of accuracy the precise point of disease. As the hour is wearing away, and I am desirous you should all have an opportunity of examining the patient with the stethescope, we will defer a further and more general consideration of these sounds, their significance, and so on, until we shall arrive at the subject in the regular curriculum of the lecture room.

*Examination of Patient.*—This was made under some disadvantages, as he was moderately under the influence of digitalis, and the sounds were somewhat obscured. *Impulse:* Muffled, and lacking in sharpness and quickness; very irregular and intermittent—beating rapidly two or three beats, intermitting a beat, and then three or four following in rapid succession. Intervals between pulsations, also, very variable. *Sounds:* the systolic and diastolic sounds are in a manner merged, by the occurrence of a rough drawing sound immediately preceding and accompanying the diastole, and diminishing the interval between the first and second sounds. This *bruit de scie* is heard more distinctly just below the level of the nipple, and across the body of the heart, diminishing over the aorta, and conveys the idea of the blood, after being propelled into the ventricle, hissing through a small opening back again—the situation corresponding to the left auriculo-ventricular opening. From the fact that the patient has had one or two attacks of hæmoptysis; from the dyspnœa and general œdema, there is evidently some pulmonary congestion; and the position and character of the *bruit* point to a thickened and indurated condition of the mitral valve, allowing the blood to regurgitate from the ventricle, and so favoring pulmonary congestion. Hypertrophy is sufficiently proved by the muffled impulse—more a swelling out under the hand, than the true shock of a natural impulse, and by percussion. Dilatation is indicated by the absence of any corresponding increase of strength in the heart's action. The œdema of the extremities results from the combined influence of impoverished blood and irregular circulation; and it often happens that the dropsical symptoms are increased by the excretion of albumen with the urine, in connection with organic disease of the heart. The prognosis, in a case of this kind, is unfavorable; and the treatment can only be palliative. The avoidance of all active muscular exercise; the use of a plain but nutritious diet; and the moderate use of diuretics with mild sedatives, will do as much to comfort the patient and prolong life, as any course that could be pursued.

